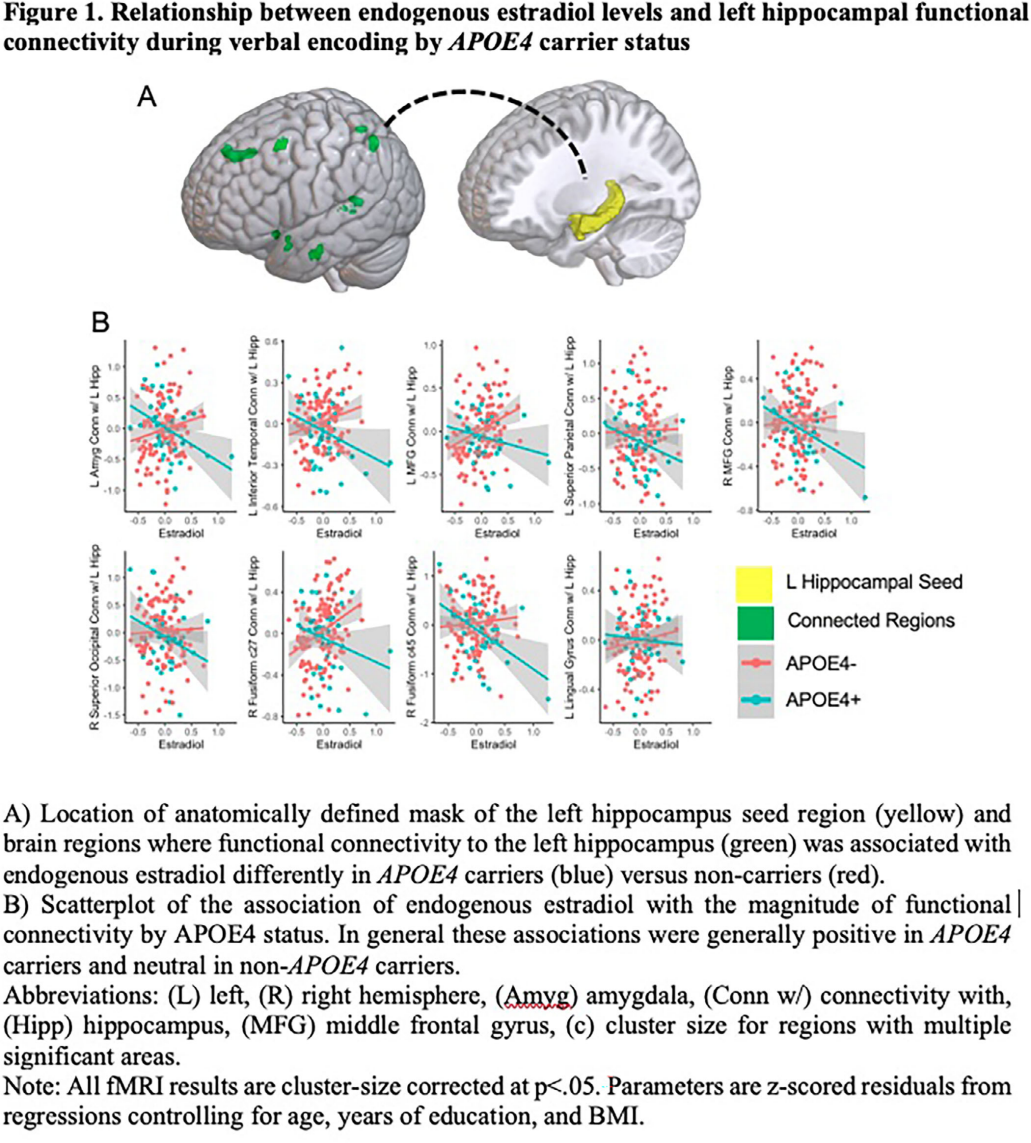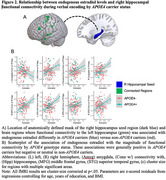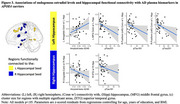# Associations of Estradiol and *APOE4* Genotype with Hippocampal Connectivity during Verbal Encoding in Postmenopausal Women

**DOI:** 10.1002/alz70856_104927

**Published:** 2026-01-11

**Authors:** Rachel A Schroeder, Rebecca C. Thurston, Minjie Wu, Howard J Aizenstein, Thomas K Karikari, M. Ilyas Kamboh, Ann D Cohen, Pauline M Maki

**Affiliations:** ^1^ University of Pittsburgh Medical Center, Pittsburgh, PA, USA; ^2^ Department of Psychiatry, School of Medicine, University of Pittsburgh, Pittsburgh, PA, USA; ^3^ University of Pittsburgh, Pittsburgh, PA, USA; ^4^ University of Pittsburgh Alzheimer's Disease Research Center (ADRC), Pittsburgh, PA, USA; ^5^ University of Pittsburgh School of Medicine, Pittsburgh, PA, USA; ^6^ Departments of Psychiatry, Psychology, and Obstetrics & Gynecology, University of Illinois Chicago, Chicago, IL, USA

## Abstract

**Background:**

High levels of endogenous estradiol in postmenopausal women are associated with beneficial effects on memory circuitry after menopause. Here we extend this work to examine 1) how estradiol and hippocampal functional connectivity (HippfConn) vary as a function of *APOE4* carrier status, and 2) whether estradiol‐associated patterns of HippfConn relate to adverse AD biomarker profiles among *APOE4+* women.

**Methods:**

Participants were enrolled in MsBrain, a cohort study of postmenopausal women, a subsample of whom completed 3T MRI neuroimaging, estradiol assessment, and plasma AD biomarker (pTau181, pTau231, amyloid‐beta 42/40) measures (*n* = 172, mean age 59.3 ± 3.9 years, 83.1% white, 23.33% *APOE4* carriers). Interactive associations of estradiol levels and *APOE4* genotype (*APOE4+* versus *APOE4‐*, excluding *APOE2*) with whole‐brain left and right HippfConn during a word encoding fMRI task were analyzed via linear regression. Next, the magnitude of association between each estradiol‐related HippfConn and AD biomarkers was examined, stratified by *APOE4* genotype. Models controlled for age, years of education, and body mass index, and were cluster‐corrected at *p* <.05.

**Results:**

*APOE4*+ status modified the association of estradiol with both left and right HippfConn during encoding. Among *APOE4*+ women, higher estradiol was associated with lower connectivity between the left HippfConn to the left inferior temporal lobe, left superior parietal lobe, right middle frontal gyrus (MFG), right fusiform, left MFG, left amygdala and parahippocampal gyrus, left lingual gyrus, and right superior occipital lobe; in contrast, in *APOE4*‐ women, those associations were beneficial or neutral (Figure 1). Among *APOE4*+ women, higher estradiol was associated with increased right HippfConn to right cingulate gyrus, right MFG, right superior temporal gyrus (STG), left STG, and left amygdala; in contrast, associations in *APOE4*‐ women were neutral or negative, except for the amygdala (Figure 2). HippfConn was more strongly related to AD pathology in *APOE4+* than *APOE4*‐ individuals (Figure 3).

**Conclusion:**

Among *APOE4*+ women, higher estradiol was associated with poorer patterns of HippfConn and increased AD pathology, as measured by plasma biomarkers. Among *APOE4‐* women, higher estradiol levels appeared beneficial for memory circuitry. These findings challenge previous assumptions that estradiol is universally beneficial to the brain, particularly for postmenopausal *APOE4+* women.